# The BRAF^V600E^ mutation: what is it really orchestrating in thyroid cancer?

**DOI:** 10.18632/oncotarget.210

**Published:** 2010-12-31

**Authors:** Carmelo Nucera, Jack Lawler, Richard Hodin, Sareh Parangi

**Affiliations:** ^1^Thyroid Cancer Research Laboratory, Endocrine Surgery Unit, Massachusetts General Hospital, Harvard Medical School, Boston, MA, USA; ^2^Division of Cancer Biology and Angiogenesis, Department of Pathology, Beth Israel Deaconess Medical Center, Harvard Medical School, Boston, MA, USA

**Keywords:** BRAFV600E, thrombospondin-1, integrins, collagen, laminin, extracellular matrix, gene set enrichment analysis, thyroid, cancer

## Abstract

BRAF^V600E^ is a constitutively active onco-kinase and is the most common genetic alteration in papillary thyroid carcinoma (PTC), and in anaplastic thyroid carcinoma as well, albeit at a lower frequency. The BRAF^V600E^ mutation in some studies has been significantly associated with extra-thyroidal extension, metastases, recurrence, and mortality in patients with PTC. A recent genome-wide expression profiling approach (Gene Set Enrichment Analysis (GSEA)) and *in vitro* and *in vivo* functional studies revealed that BRAF^V600E^ affects extracellular matrix composition (i.e. increased expression of some collagens and laminins) and promotes thyroid cancer migration and invasion. BRAF^V600E^ through the phospho-MEK1/2 and phospho-ERK1/2 pathway may control a network of genes crucial in integrating and regulating the extracellular and intracellular signaling in thyroid cancer cells, which may be fundamental to trigger an abnormal cell differentiation/totipotency and shape/polarity, and contribute to tumor aggressiveness mechanisms (i.e. cell adhesion, migration, and invasion). Increasing our knowledge of BRAF^V600E^-modulated ECM genes and targeting the subset of genes essential for tumor aggressiveness will help establish a novel paradigm for treatment of thyroid cancers harboring BRAF^V600E^. Furthermore, identifying downstream events from the BRAF^V600E^/ERK1/2 pathway will eventually identify novel biomarkers that can be used to correlate with disease outcome and overall survival.

Thyroid carcinomas of follicular cell origin represent the most frequent endocrine malignancy and include papillary (PTC), follicular (FTC), and anaplastic (ATC) types. PTC with its incidence increasing by almost 5% each year, currently ranks as the eighth most common malignancy diagnosed in women [1]. Surgical treatment, thyroid suppressive therapy and radioiodine treatment in patients at high risk for recurrence is standard [2]. Some clinical factors have been used to predict which patients with thyroid cancer might have worse prognoses. Factors such as older age (>45 years old), male gender, certain histologic subtypes (i.e. tall cell, columnar cell and diffuse sclerosing variants of PTC), tumor size greater than 4 cm and presence of extrathyroidal extension are associated with neck recurrences and distant metastases [3].

Activation of the RET/RAS/BRAF/MAPK (mitogen-activated extra-cellular signal regulated kinase) signaling pathway is the most common oncogenic event in PTC tumorigenesis. More recently certain genetic alterations in PTCs have been analyzed and some of these have also been linked to poorer outcomes. Most PTCs have either a RET/PTC translocation (10–50% of PTCs) [4,5], Ras mutations (about 12% of PTCs) [6], or the BRAF^V600E^ mutation (29–83% of PTCs) [7-9]. The V600E hotspot mutation affects the exon 15 of BRAF, a member of the RAF family of kinases, has been recently identified in PTC. BRAF^V600E^ can not only initiate tumorigenesis in normal thyroid follicular cells but is also required to maintain and promote the progression of PTC to aggressive PTC [10,11]. Of relevance, BRAF^V600E^ oncoprotein elicits elevated kinase activity that leads to activation of the MAPK (i.e. MEK1/2 and ERK1/2) signaling pathway [12-14]. Some clinical correlative studies have pointed to the increased incidence of worse histopathologic parameters such as lymphovascular invasion, extrathyroidal extension, neck lymph nodal disease, and mortality in those with BRAF^V600E^ mutation [10,15, 16]. Animal studies also point to an important role for this mutation in the aggressive behavior of PTCs; thyroid tumors from BRAF^V600E^ transgenic mice display extrathyroidal extension and foci of poorly/undifferentiated thyroid carcinoma [17]. In addition, we have recently shown that an orthotopic mouse model of ATC cells harboring BRAF^V600E^ mutation and engineered to express GFP (Green Fluorescent Protein) displays tumor aggressiveness and lymph node and lung metastases that recapitulates an advanced human thyroid cancer [18].

BRAF^V600E^ is crucially required to maintain and promote thyroid cancer progression [19]. Little information is known in PTC and ATC about which molecular targets of the BRAF^V600E^ mutation and its consequent downstream phospho-MEK1/2 and phospho-ERK1/2 kinases deregulation leads to this worse prognosis. While it is not clearly understood how BRAF^V600E^ mutation leads to more aggressive PTCs, altered genes and pathways that have been studies include: aberrant methylation of important tumor suppressor genes (i.e. TIMP-3), up-regulation of metallo-proteases-2, -3, -9 and -13 (MMP-2, -3, -9 and -13) expression [20,16], up-regulation of micro-RNAs (i.e. miR-146b) [10,21], lower expression of thyroperoxidase (TPO) and sodium iodide symporter (NIS) genes (genes important for iodine metabolism and iodine cell uptake) [22], and chromosomal instability described in rat thyroid cells [23].

We have recently investigated how BRAF^V600E^ positive PTCs differ in the expression of gene sets and found up-regulation of Extracellular Matrix (ECM) microenvironment genes in BRAF mutated PTC compared with wild type (wt) BRAF PTC or normal thyroid tissue [19]. ECM is a three-dimensional structure surrounding human cells [24]. ECM is formed of cellular and non-cellular components. The cellular component includes: endothelial and lymphatic cells, fibroblasts, immune cells (i.e. macrophages, lymphocytes), adipocytes. The non-cellular components include soluble factors such as growth factors and cytokines, and soluble (e.g. Vascular Endothelial Growth Factor (VEGF)) and insoluble proteins (e.g. Thrombospondin-1 (TSP-1), Fibronectin (FN), collagens, laminins, etc.). The normal ECM had been considered for many years as architectural scaffolding but many studies over the last decade have shown important links between genetic changes in tumor cells and post-translational modifications (glycosylation, collagens and laminins cross-linking, mechanical events such as visco-elasticity or stiffness, which have been implicated in tumorigenesis and tumor invasion) of the ECM proteins. These modifications then significantly alter tumor and stromal cellular behavior and influence gene expression. ECM remodeling in the tumor microenvironment represents a complex process able to promote tumor growth, adhesion, motility, invasion, enhance cancer cell survival, enables metastatic dissemination, and facilitate establishment of tumor cells at distant sites [24]. In addition, tumor intracellular signal transduction pathways cross-talk with the ECM cellular and non-cellular components, as well as endothelial cells themselves and may lead to angiogenesis, which is another crucial process important for tumor progression [25].

DNA microarray-based gene expression signatures underlying oncogenic pathways have been used to define cancer subtypes, recurrence of disease, and response to specific therapies. We have recently used a genome wide expression profiling analysis, GSEA (Gene Set Enrichment Analysis), to compare the genomic signature of PTCs with and without BRAF^V600E^ as well as normal thyroid tissue [19]. Our data indicated that out of 539 gene sets analyzed, 17 up-regulated and one down-regulated gene sets exclusively linked to the genomic signature of BRAF^V600E^ positive PTCs [19]. The up-regulated gene sets were mainly enriched in leading edge genes known to be important in ECM remodeling process (i.e. cell adhesion, migration, invasion, and metastasis). These 17 up-regulated gene sets included genes involved in the following biological processes: “oncogenic KRAS signaling”, “cell cycle regulation”, “cell adhesion, migration, and invasion”, “cell signaling”, “inflammatory and immune mechanisms”, “EGF receptor activation”, “cytokines mechanisms”, “estrogen-receptor-dependent signal transduction”, “vesicular intracellular trafficking machinery”, “ubiquitination mechanisms”, “caspases cascade”, and “platelet aggregation” [19]. The only down-regulated gene set included genes involved in the tyrosine metabolism and in the establishment of differentiation and polarity in epithelial cells [19].

We therefore identified new gene targets of BRAF^V600E^ in PTCs, including those involved in the regulation of tumor ECM genes expression (Figure 1). Overall, our results demonstrate that the genes listed below are positively regulated by BRAF^V600E^ and elicit important cellular signaling cross-talks, regulate ECM remodeling, and may help explain how this single gene mutation triggers thyroid tumor cell migration and invasion thus causing a worse clinical picture for patients harboring the mutaton [19].

**Figure:**
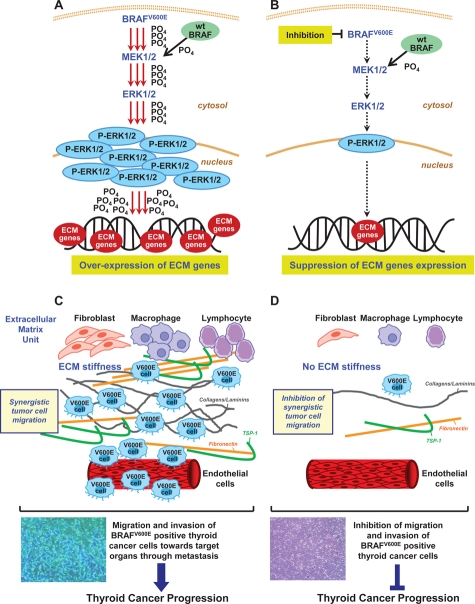
Effects of activated phospho-ERK1/2 signaling pathway by BRAF^V600E^ in human thyroid cancer cells (A) The BRAF^V600E^ oncoprotein is constitutively active kinase and does not require Ras signaling to phosphorylate MEK1/2 and ERK1/2 kinases. BRAF^V600E^ elicits a strong kinase activity (increased phosphorylation, PO4−) compared with wild type (wt) BRAF and activates MEK1/2 to phospho-MEK1/2 and ERK1/2 to phospho-ERK1/2, which may be involved in the up-regulation of some ECM remodeling genes (i.e. TSP-1, Fibronectin, cathepsins) and ECM receptors (i.e. CD44 and integrin α3, α6, and β1). ECM genes may trigger tumor cell migration and invasion, and cause thyroid cancer progression. (B) BRAF^V600E^ knockdown down-regulates phospho-MEK1/2 and phospho-ERK1/2 protein levels and might result in inhibition of thyroid cancer progression. (C) Thyroid cancer cells harboring BRAF^V600E^ show an increased ECM stiffness (e.g. collagen or laminin cross linking) and disrupts thyroid cell morphogenesis and polarity compared to normal thyroid tissue. This mechanism contributes to thyroid cancer progression by increasing cell adhesion, migration, invasion, and metastasis. (D) BRAF^V600E^ knockdown results in decreased ECM stiffness and inhibition of thyroid cancer cell migration and invasion.

Some examples of genes important in ECM regulation that were discovered to be linked to BRAF^V600E^ mutant PTCs [19] included:

## TSP-1

TSP-1 is a crucial component of the ECM and important regulator of angiogenesis. It is a multifunctional matricellular protein (structured with N- and C-terminal domains, and 3 types of sequence repeats) that regulates ECM structure during tissue genesis. TSP-1 binds to a wide variety of integrins (i.e. α3, α6, and β1 involved in cellular proliferation and metastasis pathways) and non-integrin (i.e. proteoglycans) cell surface receptors, indicating its importance in cross-talk between ECM receptors. TSP-1 together with its target TGFβ1 (that was also significantly up-regulated in BRAF mutated PTC) appear to be crucial regulator of epithelial cell growth, motility, polarity and morphogenesis [26].

Integrins (α3, α6, and β1) are the major receptors for cell adhesion to the ECM, are also known to interact with TSP-1 [27] and initiate ‘outside-in’ signal transduction events that modulate gene expression, cell proliferation, migration, and invasion [28].

## CD44

CD44 is a transmembrane receptor for hyaluronic acid (HA), which is implicated in various adhesion-dependent cellular processes, including tumor cell migration, invasion, and metastasis [29]. TSP-1 interacts with CD44 ligands or HA binding proteins to influence CD44 expression [30]. Interestingly, CD44 expression is deficient in TSP-1 null mice [30]. CD44 cleavage is mediated by TGFβ1 through the up-regulation of membrane type 1-matrix metalloproteinase and promotes migration of melanoma cells [31].

## FN

FN is another crucial ECM component, which interacts with integrins (e.g. α5β1) to regulate cytoskeleton organization, cell shape, and proliferation in human cells [32]. Fibronectin and TSP-1 can form ternary complex and regulate ECM assembly and cell adhesion [33]. There is some evidence that changes in integrin expression can result in the regulation of secretion of cathepsin-B. The 12-lipoxygenase metabolite of arachidonic acid that can up-regulates surface expression of integrins, induces secretion of cathepsin-B from tumor cells [34].

## Cathepsin-B (CTS-B) and Cathepsin–S

Cathepsin-B (CTS-B) and Cathepsin–S (CTS-S) are cysteine proteases and have been implicated in ECM remodeling, in particular in processes important for tumor development and progression, including angiogenesis, cell proliferation and invasion [35].

The main level of organization of ECM is the basement membrane that interacts directly with the epithelium and endothelium and consists primarily of collagen IV, laminins, entactin/nidogen and heparin sulphate proteoglycans. The second level of organization is the interstitial matrix, which is characterized of collagen type I and III that along with fibronectin contribute to the mechanical strength of the tissue [24]. Importantly, we found that collagen type I α1 (COL1A1) (5-fold change), collagen type III α1 (COL3A1) (2-fold change), and laminin γ2 (LAMC2) (2.45-fold change) were significantly increased in BRAF^V600E^ positive PTCs [19]. These differentially expressed genes may be related to the carcinogenesis and progression of malignant thyroid growth. These results highlight and reinforce the critical role that BRAF^V600E^ plays to up-regulate the expression of different ECM non-cellular components by altering stoichiometry of interactions between BRAF mutated tumor thyroid cells and ECM molecules, and ultimately promoting thyroid cancer cell migration and invasion (Figure 1).

As future research continues to focus on finding more selective and targeted compounds to truly personalize cancer care, researchers will have to be able to understand the molecular fingerprints and subsequent actions of each one of the mutations in common cancers. Thyroid cancer is clearly one of the most studied human cancers and many different genetic alterations have been studied. Studying how BRAF^V600E^ altered kinase activity in patients with PTC creates alterations in the already complex tumor cell–ECM interactions has been a logical next step for our laboratory. We hope that our work will lead to the development of more selective targeted anticancer therapies and also biomarkers that may include putative effectors (i.e. TSP-1) that may be measurable as secreted-factors in biologic fluids. To assay candidate biomarkers that were emerged from our study will be relevant during clinical trials (e.g. using selective inhibitors of BRAF^V600E^) in patients with BRAF^V600E^ positive PTC before treatment as well as during treatment as significant prognostic indicators of disease-free and overall survival.
